# Optimizing Wound Healing in Radial Forearm Donor Sites: A Comparative Study of Ulnar-Based Flap and Split-Thickness Skin Grafting

**DOI:** 10.3390/biomedicines13051131

**Published:** 2025-05-07

**Authors:** Adam Galazka, Katarzyna Stawarz, Karolina Bienkowska-Pluta, Monika Paszkowska, Magdalena Misiak-Galazka

**Affiliations:** 1 Head and Neck Cancer Department, Maria Sklodowska-Curie National Research Institute of Oncology, W.K.Roentgen 5, 02-781 Warsaw, Poland; 2 Maria Sklodowska-Curie Medical Academy, Evimed Medical Center Ltd., Plac Zelaznej Bramy 10, 00-136 Warsaw, Poland; 3 Department of Pathology, Maria Sklodowska-Curie National Research Institute of Oncology, W.K.Roentgen 5, 02-781 Warsaw, Poland

**Keywords:** wound healing, ulnar-based transposition flap (UBTF), split-thickness skin graft (STSG), radial forearm free flap (RFFF), tissue regeneration

## Abstract

**Background:** The radial forearm free flap (RFFF) is a common technique in head and neck reconstructive surgery. This study aimed to compare the clinical and biochemical outcomes of wound healing following ulnar-based transposition flap (UBTF) versus split-thickness skin grafting (STSG) for donor site closure, with a particular emphasis on tissue regeneration. **Materials and Methods:** A total of 24 patients (6 women, 18 men), underwent RFFF reconstruction. The donor site was closed using the UBTF technique in 10 cases, while STSG was performed in 14 cases. Postoperative complications—including necrosis, edema, hematoma, infection, and wound dehiscence—along with healing times were assessed daily during the first seven postoperative days and at monthly follow-ups over six months. Pre- and postoperative biochemical analyses included hemoglobin (HB), white blood cell count (WBC), platelets (PLT), albumin, and C-reactive protein (CRP) levels. An aesthetic evaluation of the flap was also performed. **Results:** The two groups were homogeneous. Postoperative complications occurred more frequently in the STSG group, which also demonstrated significantly longer healing times (*p* = 0.0004). In contrast, the UBTF group showed significantly better aesthetic outcomes in terms of skin color (*p* = 0.000021), skin texture (*p* = 0.000018), and flap stability (*p* = 0.0398). Additionally, pre- and postoperative PLT counts were significantly higher in the UBTF group (*p* = 0.001 and *p* = 0.043, respectively). **Conclusions**: While STSG remains a well-established method for forearm donor site closure following RFFF harvest, this study demonstrates that UBTF is a viable alternative associated with better clinical and aesthetic outcomes.

## 1. Introduction

Wound healing is a fundamental aspect of reconstructive surgery, particularly in the treatment of patients with burns, traumatic injuries, and surgical defects [1]. Its importance extends well beyond aesthetic restoration, with primary emphasis placed on functional tissue recovery and the restoration of skin integrity [2]. In parallel, regenerative medicine has emerged as a rapidly evolving field, introducing innovative approaches such as bioengineered skin substitutes, growth factor delivery, 3D-bioprinted scaffolds and cell-based therapies that are designed to enhance and accelerate the healing process [3,4,5]. As regenerative approaches continue to evolve, they promise to bridge critical gaps left by conventional therapies, ultimately advancing personalized and durable clinical solutions.

Recent innovations, such as dermal regeneration templates and autologous spray-on cell therapies, have expanded the therapeutic landscape, offering alternatives to traditional grafts by promoting vascularization and reducing donor site morbidity [6,7]. Bioengineered skin grafts, composed of biocompatible scaffolds that support cellular proliferation and tissue regeneration, are specifically designed to mimic the structure and function of native skin [8]. Conversely, spray-on cell therapies—commonly used in the treatment of acute and chronic wounds, including burns of various etiologies, diabetic and venous ulcers, post-cancer surgical wounds, and hypopigmentation disorders—offer the advantage of significantly reducing the donor site area required compared to conventional autologous skin grafting [9,10]. In addition, advances in skin 3D bioprinting, which uses bioprintable materials known as bioinks—including natural polymers such as collagen, alginate, chitosan, hyaluronic acid, and cellulose—have introduced new possibilities in regenerative medicine [11,12]. This technology enables precise spatial control over the distribution of different skin components, allowing for the fabrication of complex structures such as the epidermis, dermis, and vascular networks [12]. Furthermore, 3D bioprinting holds the potential for personalized wound coverage, offering customized and more effective treatment options for complex skin defects [13].

However, despite these advancements, traditional clinical techniques, including autologous skin grafting and flap reconstruction, remain essential in surgical practice, offering biologically compatible and clinically validated methods for effective wound closure and long-term tissue regeneration [6,14,15].

The current practice of reconstructive surgery and wound care still relies heavily on skin grafting techniques and flap-based reconstruction [16,17]. Although often associated with plastic and aesthetic surgery, these techniques are equally vital in surgical oncology, particularly in reconstructions following breast cancer treatment and head and neck cancer resections [18,19].

One of the fasciocutaneous flaps most widely used in head and neck reconstruction is the radial forearm free flap (RFFF), especially for defects involving the tongue and oral cavity [20,21]. Its versatility and high survival rate—exceeding 95%—make it an excellent choice for addressing a wide range of complex defects in this region [22]. However, despite its effectiveness, donor site closure remains a significant clinical challenge, often accompanied by complications such as partial or total graft necrosis, infection, and delayed healing [23,24]. A variety of closure techniques have been described in the literature, including direct closure, local flaps, skin grafting, tissue expansion, and the use of artificial dermal substitutes [25,26,27]. Among these, skin grafting remains the most commonly employed method; however, it is frequently associated with suboptimal aesthetic and functional outcomes and prolonged healing times [28].

Numerous modifications of skin grafting have been proposed, including the use of both split-thickness (STSG) and full-thickness skin grafts (FTSG) [27,29]. In a study by Al-Aroomi et al., STSG and FTSG were compared for donor site closure. While functional outcomes were similar, FTSG resulted in superior aesthetic results [30]. However, their evaluation was limited to clinical complications, without addressing biochemical or regenerative parameters. Similarly, a study by Mashrah et al. evaluated the use of a bilobed flap for donor site reconstruction but focused solely on functional outcomes, omitting an analysis of biological or regenerative aspects [31].

An alternative technique described in the literature is the ulnar-based transposition flap (UBTF), which presents a promising solution for forearm donor site closure [32]. This method may allow for shorter healing times and avoids the need for tissue harvest from a secondary site, potentially reducing overall morbidity [33]. However, existing studies primarily assess aesthetic and functional outcomes, with limited investigation into biochemical or regenerative markers of tissue healing.

Therefore, the objective of the present study was to evaluate and compare donor site morbidity, healing times, aesthetic outcomes, and biochemical markers of wound healing between patients undergoing RFFF donor site closure with either the UBTF or STGS. This single-institution clinical experience aims to contribute novel insights into optimizing donor site management strategies, integrating both clinical and regenerative perspectives in head and neck reconstruction.

## 2. Materials and Methods

### 2.1. Subjects

We conducted a monocentric retrospective case-control study from January 2024 to January 2025 at the Head and Neck Cancer Department of the Maria Sklodowska-Curie National Research Institute of Oncology in Warsaw, Poland. The choice between STSG and UBTF was determined based on the size and precise location of the cancerous lesion. The study included 24 patients, with 10 undergoing the ulnar-based closure technique and 14 receiving STSG. Patient sociodemographic data were extracted from medical records using patient identifiers and hospital numbers. Pre-existing factors such as age, gender, smoking history, diabetes mellitus (DM), and atherosclerosis were documented. Additionally, clinical and pathological tumor characteristics including tumor location, histological type, and the size of the harvested RFFF were evaluated. Subsequently, a detailed analysis was performed on post-surgical complications based on follow-up data and potential contributing patient features, aimed at assessing flap morbidity. The following criteria were used in patient selection:

The inclusion criteria were:

- Patients aged ≥ 18 years.
- Histologically confirmed diagnosis of head and neck cancer requiring RFFF reconstruction.
- No contraindications for microsurgical reconstruction.

The exclusion criteria were:

- Severe systemic comorbidities contraindicating free flap surgery, including uncontrolled DM or severe peripheral vascular disease.
- Prior surgery or radiotherapy to the forearm affecting flap harvest feasibility.
- Incomplete medical records or lack of postoperative follow-up.

### 2.2. Biochemical Analysis 

Several blood parameters with potential relevance to wound healing were assessed preoperatively and one day after surgery. We evaluated white blood cell (WBC) count, which is a key indicator of immune function and may reflect the presence of infection or inflammation, both of which can adversely affect the wound healing process. Hemoglobin (HB), which plays a critical role in oxygen transport to tissues, was also measured; decreased levels may impair tissue oxygenation and delay healing. Platelet (PLT) count was evaluated as well, since PLTs are essential for clot formation and the initiation of tissue repair, making their levels important for assessing the body’s capacity to initiate healing. In addition, albumin was measured as a well-established marker of nutritional and inflammatory status; reduced albumin levels are associated with poor wound healing and an increased risk of postoperative complications. Finally, C-reactive protein (CRP)—measured only in the postoperative period—is a sensitive biomarker of systemic inflammation and may assist in the early identification of infection or an excessive inflammatory response. Blood samples were collected preoperatively from a peripheral venous access using standard procedures, and postoperatively from a central venous line. No complications were reported during the sample collection process.

### 2.3. Flap Morbidity Assessment 

Flap assessments were conducted daily during the initial 7-day postoperative hospital stay, with a focus on identifying potential complications. Each flap was evaluated three times per day. The assessed complications included edema, flap necrosis, wound dehiscence, hematoma formation, and infection. Each variable was scored on a binary scale: 0, indicating normal findings, and 1, indicating the presence of a confirmed complication. The scores were then summarized and reported as mean values with corresponding standard deviations (SD). Follow-up evaluations were subsequently performed postoperatively at regular one-month intervals throughout the first six months. Additionally, healing time was assessed for patients in both groups using the following formula:

*Healing Time* (days) = *Date of Complete Healing* − *Date of Surgery*.

### 2.4. Aesthetic Flap Evaluation 

An aesthetic evaluation of all donor sites was conducted during the 7-day postoperative hospital stay and at monthly clinical follow-up appointments. The assessment included skin color, skin texture, and flap stability, which were compared to the surrounding tissue. Each parameter was rated on a three-point scale, where 1 indicated a poor match, 2 represented a moderate match, and 3 signified an excellent match. The scores for each evaluated variable within both groups were summarized and presented as mean values with corresponding SD.

### 2.5. Surgical Technique 

**Figure 1:**
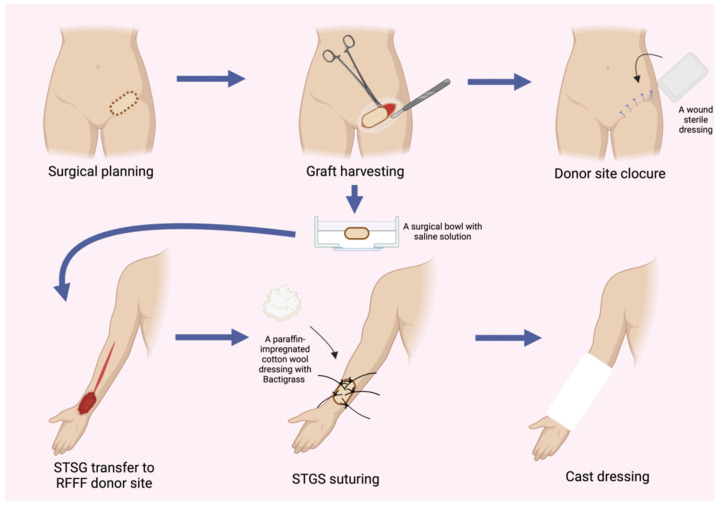
Schematic representation illustrating the step-by-step procedure for harvesting an STSG and subsequent closure of the RFFF donor site. Abbreviations: RFFF—radial forearm free flap; STSG—split-thickness skin graft.

**Figure 2:**
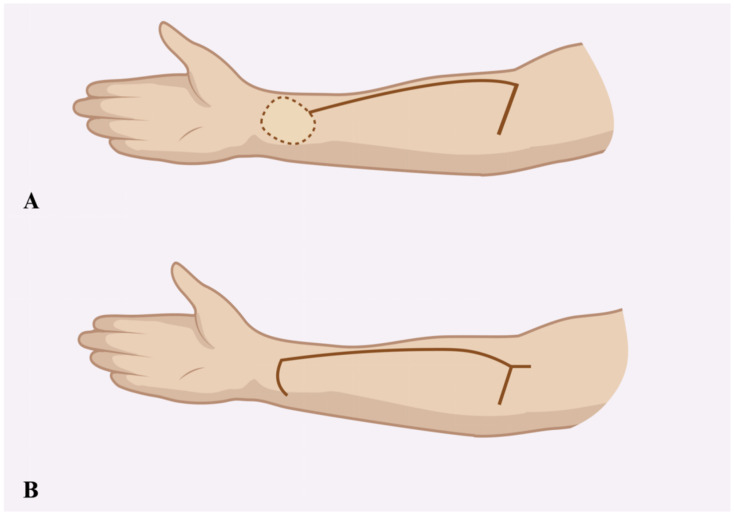
Schematic illustration showing the incision design for the ulnar-based transposition flap (**A**) and the typical appearance of donor site closure following flap transfer (**B**).

**Figure 3:**
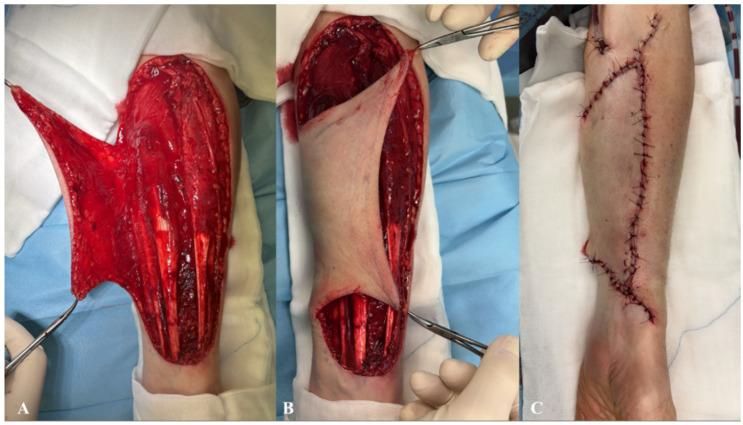
Schematic intra-operative illustration of the ulnar-based transposition flap technique. (**A**) Elevated view of the ulnar-based flap; (**B**) flap transposition to cover the radial forearm free flap (RFFF) donor site; (**C**) final closure with skin sutures and insertion of a drainage tube.

The donor site following the RFFF harvest was closed using two different techniques. The first method involved harvesting an STSG from the patient’s groin using a size 20 blade. The harvest site was pre-marked with a surgical marker after measuring the donor site dimensions with a surgical ruler. An incision was then made with a size 20 blade, and the graft was carefully elevated with minimal traction until fully excised. The donor site was subsequently sutured using only Dermalon skin suture (3-0) (Covidien Ilc^®^, Mansfield, MA, USA) (Figure 1). The ulnar-based transposition flap technique (Figure 2) began with an incision along the ulnar side of the forearm, carefully preserving the subcutaneous tissue. The flap was then elevated with minimal tension and rotated to cover the RFFF donor site. A 16.0 surgical drain (B.Braun^®^, Melsungen, Germany) was inserted, and the flap was secured using interrupted cutaneous and subcutaneous sutures (3-0) (Covidien Ilc^®^, Mansfield, MA, USA) (Figure 3). All patients received postoperative prophylaxis with Enoxaparin sodium (40 mg subcutaneously) once daily for seven days during their post-surgical hospitalization. Additionally, the standard intravenous antibiotic regimen consisted of 2 g of Ceftriaxone and 1.5 g of Metronidazole, administered intravenously on a daily basis for four days post-operation.

### 2.6. Statistical Analysis

Categorical variables were presented as frequencies and percentages and compared between groups using the Chi-square test or Fisher’s exact test as appropriate. Continuous variables were expressed as means with SD, and group comparisons were conducted using either the independent samples *t*-test or the Mann–Whitney U test depending on data distribution. All statistical analyses were performed using RStudio (Version 1.4.1564) on macOS 10.15.7. A *p*-value < 0.05 was considered statistically significant.

## 3. Results

### 3.1. Patients Characteristics 

**Figure 4:**
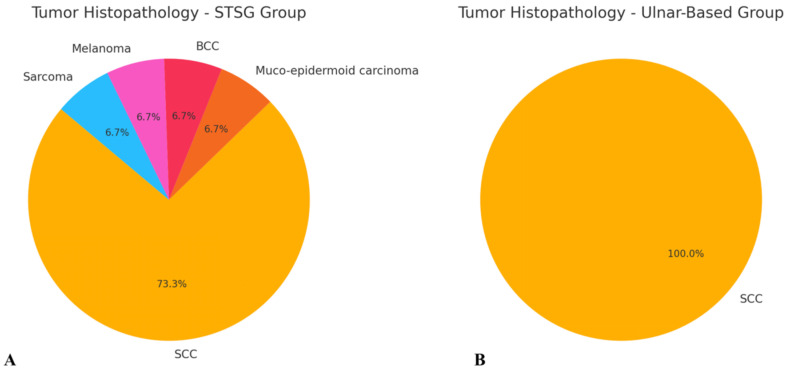
Schematic representation of tumor histopathology in both evaluated groups: (**A**) STSG group and (**B**) ulnar-based flap group. Abbreviations: BCC—basal cell carcinoma; SCC—squamous cell carcinoma.

**Figure 5:**
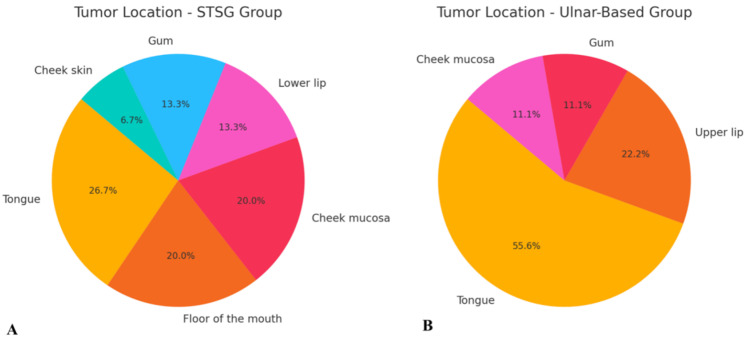
Schematic representation of tumor location distribution in both evaluated groups: (**A**) STSG group and (**B**) ulnar-based flap group. Abbreviation: STSG—split-thickness skin graft.

A total of 24 patients (18 males and 6 females) diagnosed with head and neck cancer were included in the study. The majority of cases (20 patients) were diagnosed with squamous cell carcinoma (SCC), while one patient was diagnosed with mucoepidermoid carcinoma, one with melanoma, one with sarcoma, and one with basal cell carcinoma (BCC) (Figure 4). The most common tumor location within the head and neck region was the tongue (9 patients, 37.5%), followed by the cheek mucosa (4 patients, 16.7%) (Figure 5). Patients were divided into two groups: the STGS group (14 patients, 62.3%) and the UBTF group (10 patients, 41.7%). The mean age of patients in the UBTF group was 57.8 ± 14.1 years, while in the STSG group, it was 63.2 ± 13.8 years. There were no significant differences between the two groups in terms of gender, age, tumor histopathology, tumor location, smoking history, DM2 or atherosclerosis, and the size of harvested RFFF. A detailed overview of patient demographics and clinical characteristics is presented in Table 1.

### 3.2. Biochemical Results Evaluation 

The biochemical analysis was conducted using the mean values of HB, WBC count, PLT count, and albumin before and after surgery. Among these parameters, the only statistically significant difference between the UBTF and STGS groups was observed in PLT levels, both preoperatively (*p* = 0.0018) and postoperatively (*p* = 0.0439) (Table 2). Additionally, postoperative CRP evaluation revealed higher CRP levels in the STSG group compared to the UBTF group (64.28 mg/L vs. 47.58 mg/L, respectively), although the difference was not statistically significant (*p* = 0.747).

### 3.3. Aesthetic Flap Assessment 

**Figure 6:**
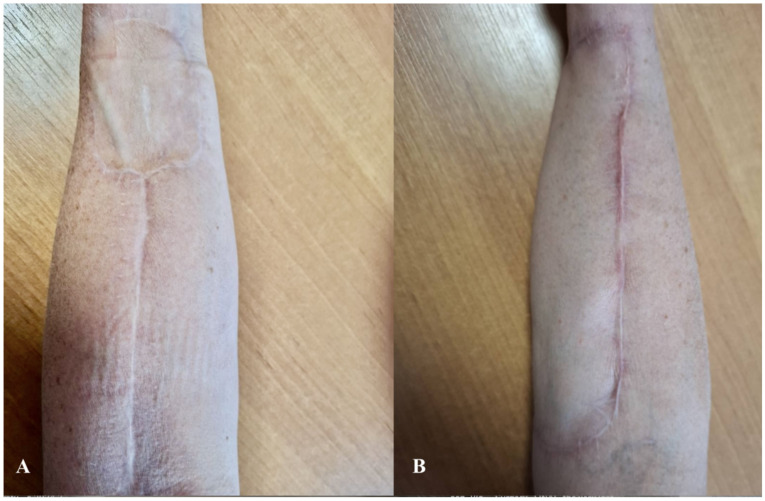
Schematic representation of aesthetic outcomes three months post-surgery using (**A**) the STSG technique and (**B**) the UBTF technique. Abbreviations: STSG—split-thickness skin graft; UBTF—ulnar-based transposition flap.

The analysis revealed that, in terms of aesthetic flap assessment, all evaluated parameters—including skin color, skin texture, and flap stability—were significantly better in the UBTF group compared to the STSG group. The most pronounced differences were observed in skin color (*p* = 0.000021) and skin texture (*p* = 0.000018), where the UBTF flap group demonstrated superior aesthetic outcomes (Table 3). A postoperative follow-up image taken three months after surgery demonstrated satisfactory aesthetic outcomes following both the STSG and UBTF techniques (Figure 6).

### 3.4. Post-Surgical Complications Assessment 

The analysis of post-surgical complications revealed no cases of flap necrosis in either group. However, edema, infection, and wound dehiscence were observed more frequently in the STSG group compared to the UBTF group. Despite this trend, the differences were not statistically significant (*p* > 0.05). In contrast, the evaluation of healing times demonstrated a significantly shorter healing duration in the UBTF group compared to the STSG group (*p* = 0.0004), indicating a clear advantage in regenerative efficiency (Table 4). Additionally, the analysis did not reveal any significant correlation between patient comorbidities and postoperative complications (Table 5).

## 4. Discussion

Efficient, rapid, and complication-free wound healing is a central goal of regenerative medicine. Optimizing the healing process—particularly by reducing healing time—not only accelerates overall patient recovery but also reduces healthcare costs and enhances healthcare system efficiency [34,35]. By leveraging the body’s intrinsic regenerative capacity—often via biologically active materials, growth factor delivery, or stem cell-based therapies—regenerative approaches aim to restore tissue structure and function more effectively than traditional methods [36,37].

In the era of regenerative medicine, a wide array of wound dressings, including artificial skin substitutes, has been developed. Although artificial wound dressings, such as synthetic skin substitutes and bioengineered scaffolds, have advanced significantly and contribute to wound healing by promoting moisture retention, infection control, and even cellular stimulation, they typically serve as temporary covers that facilitate secondary intention healing rather than providing immediate, definitive tissue replacement [38,39,40]. In contrast, skin grafting techniques such as STSG remain the gold standard for donor site closure following RFFF harvest. STSG offers definitive coverage, biological integration, and durable mechanical strength, qualities that artificial dressings have yet to fully replicate [41]. Moreover, skin grafts achieve full dermal–epidermal barrier restoration and are particularly suitable for covering larger or surgically created defects like RFFF donor sites, where rapid, stable closure is essential to minimize morbidity and functional impairment.

Therefore, while advanced technologies in tissue healing are increasingly adopted in daily clinical practice, surgical techniques focused on minimizing healing time still remain foundational to successful patient care [42]. The synergistic integration of regenerative technologies with time-tested surgical approaches holds the greatest promise for optimizing both functional recovery and cost-effective treatment, thereby advancing standards in modern wound management.

Given the high risk of complications and morbidity associated with head and neck cancer reconstructive surgery, approaches that promote faster healing are critically important [43]. In this study, we evaluated STSG and UBTF as two distinct strategies for donor site closure following RFFF harvest. Although a range of closure techniques have been described in the literature, STSG remains the most commonly used method [44]. The main advantage of STSG lies in its ability to be precisely tailored to the donor site dimensions; however, it requires advanced surgical skills and creates an additional donor site wound [45]. Conversely, UBTF is relatively easy to perform but may be unsuitable for larger defects where the flap may not fully cover the donor area [46].

**Figure 7:**
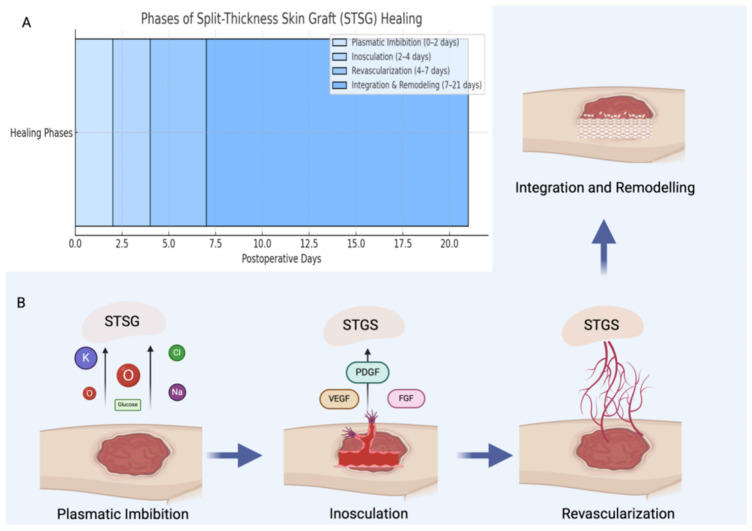
Schematic illustration showing the approximate timeline of the STSG healing process (**A**), followed by a step-by-step depiction of the biological phases involved (**B**). The process begins with plasmatic imbibition, during which basic nutrients and oxygen diffuse passively from the wound bed into the graft. This is followed by the phase of inosculation, where initial vascular connections begin to form between the graft and the recipient site. Subsequently, revascularization occurs, marked by active angiogenesis that restores a functional blood supply. Finally, the healing process concludes with integration and remodeling, during which the grafted tissue matures and reorganizes structurally to achieve long-term stability and functionality. Abbreviations: STSG—split-thickness skin graft; PDGF—platelet-derived growth factor; VEGF—vascular endothelial growth factor; FGF—fibroblast growth factor.

Our findings showed comparable postoperative complication rates between the two groups, with no instances of flap failure and no influence of patient comorbidities on healing outcomes. However, healing times were significantly shorter in the UBTF group. Despite requiring an additional incision, UBTF demonstrated favorable wound healing, likely due to its reliable vascular supply [32,47]. In contrast, STSG healing is typically slower and carries a higher risk of complications due to its reliance on a multi-phase biological process involving plasmatic imbibition, inosculation, and revascularization [48,49] (Figure 7). During the plasmatic imbibition phase, essential nutrients—including oxygen, glucose, and electrolytes—passively diffuse from the wound bed into the skin graft to maintain cellular viability. This is followed by the inosculation phase, during which initial vascular connections begin to form between the graft and the recipient site. Subsequently, revascularization occurs, characterized by active angiogenesis and the establishment of a functional microvascular network. Ultimately, the healing process culminates in tissue integration and remodeling, leading to the restoration of structural and functional integrity [50,51]. The results of our study are also consistent with those reported by Loeffelbein et al., reinforcing the regenerative advantages of vascularized local flaps over grafts for donor site reconstruction [52].

Importantly, the factors influencing wound healing extend beyond flap design. Our biochemical analysis found no significant differences in HB, WBC, or albumin levels between the two groups. Low infection rates may explain the only slightly elevated WBC values, likely attributed to the surgical intervention itself. However, PLT counts were significantly higher in the UBTF group. Given the role of PLT in clot formation and angiogenesis—mediated by the release of PDGF, VEGF, and FGF—their elevated levels may contribute to enhanced neovascularization and the reduced healing times observed in the UBTF group [53]. Lacci et al. emphasized the growing role of platelet-rich preparations in enhancing wound closure [54], while Margolis et al. demonstrated the superior efficacy of platelet releasate over standard care in treating diabetic foot ulcers [55]. High PLT counts may therefore represent a biomarker of regenerative capacity, potentially guiding flap selection in surgical planning.

The aesthetic outcomes further favored the UBTF group, which showed superior skin color, texture, and flap stability. These improvements are likely due to the use of native forearm skin, which better matches surrounding tissue. In contrast, STSG is typically harvested from other anatomical areas such as the groin, thigh, or abdomen [56], where differences in texture and pigmentation can result in less favorable aesthetic outcomes. The superior flap stability observed in the UBTF group may also be attributed to the use of tissue with similar biomechanical properties and shorter healing durations.

The primary limitations of this study include its small sample size and relatively short follow-up period. Nevertheless, most major postoperative complications occur within the first few days following surgery, aligning with the timeline of this study. Future research should involve larger cohorts and deeper investigations into the molecular and biochemical mechanisms underlying wound healing.

This study lays important groundwork and provides novel insights into regenerative and surgical approaches for donor site closure following RFFF harvest. Elevated PLT levels may serve as a predictive marker of wound healing efficacy. Compared to the widely used STSG method, UBTF demonstrates significant advantages in healing time, aesthetic outcomes, and potential regenerative benefits, establishing it as a compelling alternative in clinical practice.

## 5. Conclusions

Regenerative medicine plays a pivotal role in advancing modern clinical practice by enhancing tissue repair strategies. This study demonstrates that the use of UBTF offers significant advantages over STSG for donor site closure following RFFF harvest. Specifically, UBTF was associated with shorter healing times, superior aesthetic outcomes (including better skin color, texture, and flap stability), and a lower incidence of postoperative complications. Additionally, elevated PLT levels in the UBTF group may have contributed to enhanced angiogenesis and improved wound healing. These findings suggest that the UBTF technique may represent a superior and more efficient alternative to traditional STSG closure methods. Nevertheless, further studies with larger patient cohorts and molecular-level evaluations are warranted to validate these promising results and to refine regenerative surgical strategies for clinical application.

## Figures and Tables

**Table 1:** Patients characteristic.

Variable	UBTF (*n* = 10)	STGS (*n* = 14)	*p*-Value
Gender			0.338 ^a^
Male	6 (60%)	12 (85.7%)	
Female	4 (40%)	2 (14.3%)	
Age (mean ± SD)	57.8 ± 14.1	63.2 ± 13.8	4.521 ^b^
Primary tumor location			0.238 ^a^
Tongue	5 (50%)	4 (28.6%)	
Cheek mucosa	2 (20%)	2 (14.3%)	
Floor of the mouth	0	3 (21.4%)	
Gum	1 (10%)	2 (14.3%)	
Upper lip	2 (20%)	0	
Lower lip	0	2 (14.3%)	
Cheek skin	0	1 (7.15%)	
Histopathological Type			0.416 ^a^
SCC	9 (90%)	12 (85.8%)	
BCC	0	1 (7.1%)	
Sarcoma	1 (10%)	0	
Melanoma	0	1 (7.1%)	
Smoking history	5 (50%)	11 (78.6%)	0.305 ^a^
DM2	1 (10%)	3 (21.4%)	0.853 ^a^
Atherosclerosis	5 (50%)	8 (57.1%)	1.0 ^a^
RFFF size (cm^2^)	41.4 ± 3.503 (mean ± SD)	41.3 ± 2.225 (mean ± SD)	0.933

^a^ Chi-Square Test; ^b^ *T*-Test.

**Table 2:** Biochemical parameters analysis.

Parameter	STSG Pre-Mean ± SD	STSG Post-Mean ± SD	UBTF Pre-Mean ± SD	UBTF Post-Mean ± SD	*p*-Value (Pre-Op)	*p*-Value (Post-Op)
HB (g/dL)	13.6 ± 1.89	10.4 ± 1.41	14.5 ± 1.04	11.0 ± 1.42	0.140	0.296
WBC (×10⁹/L)	7.05 ± 2.32	11.6 ± 4.53	8.05 ± 3.77	11.9 ± 4.24	0.468	0.838
PLT (×10⁹/L)	190.2 ± 54.56	175.6 ± 55.78	280.0 ± 62.51	217.9 ± 38.60	0.001	0.0438
Albumin (mg/dL)	42.9 ± 2.87	37.1 ± 7.13	41.5 ± 2.18	34.3 ± 4.59	0.205	0.258

Abbreviations: SD—standard deviation; STGS—split-thickness skin graft; YBTF—ulnar-based transposition flap; HB—hemoglobin WBC—white blood cells; PLT—platelets.

**Table 3:** Aesthetic flap assessment.

Variable	UBTF (Mean ± SD)	STGS (Mean ± SD)	*p*-Value ^a^
Skin color	3.00 ± 0.00	1.40 ± 0.507	*p* = 0.000021
Skin texture	3.00 ± 0.00	1.67 ± 0.487	*p* = 0.000018
Flap stability	2.56 ± 0.527	1.93 ± 0.703	*p* = 0.0398

^a^ Mann–Whitney U test. Abbreviations: SD—standard deviation; STGS—split-thickness skin graft; UBTF—ulnar-based transposition flap.

**Table 4:** Post-surgical complications analysis.

Complication	UBTF Group (*n* = 10)	STSG Group (*n* = 14)	*p*-Value ^a^
Edema	5 (50%)	10 (71.4%)	0.521
Hematoma	1 (10%)	0	0.862
Infection	3 (30%)	6 (42.8%)	0.830
Wound dehiscence	2 (20%)	6 (42.8%)	0.464
Healing time (days)	15.7 ± 3.354 (mean ± SD)	21.6 ± 2.261 (mean ± SD)	0.0004

^a^ Fisher test.

**Table 5:** Analysis of patient comorbidities in relation to postoperative complications.

Comorbidity	Complication	*p*-Value ^a^
DM2	Edema	1
	Hematoma	1
	Infection	1
	Wound dehiscence	0.654
Smoking	Edema	1
	Hematoma	1
	Infection	0.654
	Wound dehiscence	1
Atherosclerosis	Edema	1
	Hematoma	0.931
	Infection	0.750
	Wound dehiscence	0.222

^a^ Fisher test.

## Data Availability

All data supporting the findings of this study are available upon reasonable request from the corresponding author.

## Abbreviations

The following abbreviations are used in this manuscript:

BCC	Basal cell carcinoma
CRP	C-reactive protein
DM2	Diabetes mellitus type 2
FGF	Fibroblast growth factor
FTSG	Full-thickness skin graft
HB	Hemoglobin
PDGF	Platelet-derived growth factor
PLT	Platelets
RFFF	Radial free forearm flap
SCC	Squamous cell carcinoma
STGS	Split-thickness skin graft
UBTF	Ulnar-based transposition flap
VEGF	Vascular endothelial growth factor
WBC	White blood cells
